# 中国成人自身免疫性溶血性贫血诊疗指南（2023年版）

**DOI:** 10.3760/cma.j.issn.0253-2727.2023.01.003

**Published:** 2023-01

**Authors:** 中华医学会血液学分会红细胞疾病（贫血）学组

自身免疫性溶血性贫血（autoimmune hemolytic anemia, AIHA）是由于机体免疫功能紊乱、产生自身抗体、红细胞破坏加速（溶血）超过骨髓代偿时发生的贫血。目前国内尚无AIHA流行病学的数据，国外资料显示AIHA的年发病率为（0.8～3.0）/10万[1]–[2]。其发病机制尚未完全明确。自身抗体的产生涉及免疫系统的多个环节：①内源性红细胞和外源性/环境抗原的交叉反应而产生的分子模拟（交叉抗原和整合抗原）；②受后天因素（感染、恶性肿瘤、药物等）影响，自身抗原结构改变（突变抗原和错误抗原），抗原呈递失调，从而产生自身抗体；③B细胞和T细胞功能障碍，包括调节性T细胞数量减少和其他T细胞异常，常见于免疫缺陷病、自身免疫病和淋巴增殖性疾病[2]。④病原微生物的线粒体DNA可以与红细胞膜表面TLR9结合，改变膜结构，降低CD47表达，激活红细胞吞噬程序，并激活固有免疫反应[3]。

为进一步规范和提高我国AIHA的诊治水平，中华医学会血液学分会红细胞疾病（贫血）学组在国内外AIHA诊治指南和共识[4]–[7]的基础上，制订本指南。

一、AIHA诊断标准和分型

1. 诊断标准：①血红蛋白水平达贫血标准；②血结合珠蛋白降低（<250 mg/L）、血总胆红素升高（≥17.1 µmol/L，以非结合胆红素升高为主）、血乳酸脱氢酶升高且网织红细胞百分比>4％或绝对值>120×10^9^/L；③检测到红细胞自身抗体。

2. 分型：

（1）依据病因明确与否，分为继发性和原发性两类。

（2）依据自身抗体与红细胞结合所需的最适温度分为温抗体型（wAIHA）、冷抗体型（cAIHA）和温冷抗体混合型（mAIHA）。

①wAIHA：自身抗体为IgG型和（或）C3d型（极少数情况下为IgA型），冷抗体阴性或弱阳性（<1∶32）。

②cAIHA：包括冷凝集素病（cold agglutinin disease, CAD）、冷凝集素综合征（cold agglutinin syndrome, CAS）及阵发性冷性血红蛋白尿症（paroxysmal cold hemoglobinuria, PCH）。CAD：自身抗体为C3d型（IgG型阴性或者弱阳性），并且冷凝激素（CA）≥64（外周血或骨髓可以存在克隆性B淋巴细胞增殖，但没有恶性肿瘤的相关临床症状，影像学没有恶性肿瘤的证据）。CAS：自身抗体为C3d型（IgG型阴性或者弱阳性），并且CA≥64。患者存在明确相关疾病，如感染、自身免疫病、B细胞淋巴瘤（伴有临床症状或影像学异常）或其他肿瘤。PCH：自身抗体为Donath-Landsteiner型。

③mAIHA：温抗体和冷抗体均阳性。

（3）依据红细胞自身抗体检测结果，分为自身抗体阳性型和自身抗体阴性型。自身抗体阴性型AIHA临床符合溶血性贫血，除外其他溶血性贫血而免疫抑制治疗有效。

二、AIHA特异性检查和常规检查

1. 特异性检查：

（1）红细胞自身抗体检查：①直接抗人球蛋白试验（direct antiglobulin test, DAT）检测被覆红细胞膜自身抗体，温抗体自身抗体与红细胞最佳结合温度为37 °C，冷抗体自身抗体与红细胞最佳结合温度为0～5 °C。②间接抗人球蛋白试验（indirect antiglobulin test, IAT）检测血清中的游离抗红细胞膜抗体。③CA试验检测血清中CA，CA是IgM型冷抗体，与红细胞最佳结合温度为0～5 °C。④冷热溶血试验检测冷热双相溶血素（D-L抗体），D-L抗体是IgG型冷热溶血素，在0～4 °C时与红细胞结合，并吸附补体，但并不溶血；在30～37 °C发生溶血。

（2）病因学检查：无基础疾病者诊断为原发性AIHA，有基础疾病则为继发性AIHA（表1）。

**表1:** 继发性自身免疫性溶血性贫血常见病因

淋巴细胞增殖性疾病
慢性淋巴细胞白血病
非霍奇金淋巴瘤
意义未明的单克隆IgM丙种球蛋白血症
霍奇金淋巴瘤
自身免疫性淋巴细胞增生综合征
实体瘤/卵巢皮样囊肿
自身免疫性疾病
系统性红斑狼疮、抗磷脂综合征、干燥综合征、类风湿关节炎
桥本甲状腺炎
溃疡性结肠炎、自身免疫性肝炎
感染
肺炎支原体
病毒感染：EB病毒、巨细胞病毒、细小病毒B19、HIV、肝炎病毒、轮状病毒、腺病毒感染、呼吸道合胞病毒和流感病毒
细菌感染
免疫缺陷
常见变异型免疫缺陷病
原发性联合免疫缺陷病
药物
嘌呤类似物：氟达拉滨、克拉屈滨
头孢菌素：头孢双硫唑甲氧、头孢曲松
哌拉西林
β-内酰胺酶抑制剂：他唑巴坦、舒巴坦
血型不合
血型不合的异基因造血干细胞移植/实体器官移植
同种免疫
输血后慢性溶血

2. 常规检测项目：

（1）血常规及分类、血涂片、网织红细胞计数、肝肾功能（含胆红素及分类）、乳酸脱氢酶。

（2）免疫全项（含抗核抗体，抗dsDNA抗体和IgG、IgA和IgM）、风湿抗体（含类风湿因子）、抗磷脂抗体、血清蛋白电泳、免疫固定电泳。有条件单位可检测B、T细胞亚群及相关细胞因子。

（3）HBV、HCV、HIV、EB病毒（EBV）、巨细胞病毒（CMV）、细小病毒B19和肺炎支原体。

（4）全身浅表淋巴结B超、胸部、腹部、盆腔CT。

（5）骨髓细胞形态学、骨髓病理、淋巴细胞免疫表型。

三、AIHA治疗

治疗指征：症状性贫血。如无特殊情况，推荐HGB<100 g/L进行治疗。

AIHA的治疗主要分对症支持治疗（包括成分输血、清除溶血产物、保护重要脏器、支持造血、控制危险因素如感染和血栓等）和控制溶血治疗（糖皮质激素、免疫抑制剂、化疗、靶点治疗、细胞治疗等）两大部分。继发性AIHA的治疗还应包括原发病的治疗。

1. 对症支持治疗：

（1）红细胞成分输血：①应尽量避免或减少输血。AIHA由于存在自身抗体，交叉配血难度增加，同种抗体致溶血性输血反应的危险增大。②输血时机应根据贫血程度、有无明显症状、发生快慢而定。对于急性溶血性贫血患者，出现严重症状时能排除同种抗体者须立刻输注红细胞。对于慢性贫血患者，HGB在70 g/L以上可不必输血；HGB在50～70 g/L时如有不能耐受的症状可适当输血；HGB在50 g/L以下时应输血。③抢救时不强调应用洗涤红细胞。④cAIHA患者红细胞输注时应注意保温。

配血困难情况的处理：检测自身抗体抗ABO、Rh血型特异性，对供者进行选择及交叉配血试验。交叉配血不完全相合时，选用多份标本交叉配血中反应最弱的输注。缓慢滴注，密切观察有无输血反应。输血前加用糖皮质激素可减少输血反应的发生并减轻输血反应程度。

（2）清除溶血产物和保护重要脏器功能：碱化利尿、利胆去黄，并注意电解质平衡。急性重度溶血发作常规治疗效果欠佳可行血浆置换术（条件允许情况下，推荐首选白蛋白）。对于cAIHA应注意置换液体的保温（有条件者可使用专业恒温设备）。

（3）支持造血：促红细胞生成素（EPO）能促进红系造血恢复，升高血红蛋白水平，改善贫血。尤其是网织红细胞正常或者减低、血EPO水平正常或轻度升高者，EPO疗效较好[8]。合并血栓者慎用。雄激素可以促进内源性EPO的产生和释放，也可选择性应用。

（4）感染的预防和治疗：AIHA自身免疫紊乱、糖皮质激素和免疫抑制剂治疗、脾切除等都会明显增加感染（尤其是致命重度感染）的概率。急性溶血发作、脾切除、长期重度免疫抑制患者应注意感染的预防。有条件者可以接种相关疫苗。一旦感染，应积极寻找感染灶和病原体，有针对性抗感染治疗。

（5）血栓的预防：11％～20％ AIHA患者发生血栓，血栓事件发生率明显高于年龄性别匹配的正常人群（*aHR*＝6.3）[9]，包括肺栓塞、深静脉血栓、脾栓塞、脑卒中和心肌梗死等。血栓的危险因素包括急性溶血发作、卧床、发热、高龄、既往血栓病史、易栓症、创伤或外科手术、呼吸衰竭、心力衰竭感染等。合并高危因素患者常规应用抗凝治疗预防血栓的发生，常用药物包括低分子肝素或口服抗凝药。

2. wAIHA的治疗（图1）：

（1）一线治疗：糖皮质激素±利妥昔单抗。

①糖皮质激素：推荐在无糖皮质激素使用禁忌情况下应用。按泼尼松计算，剂量为0.5～1.5 mg·kg^−1^·d^−1^，可以根据具体情况换算为地塞米松、甲泼尼龙等静脉输注。糖皮质激素用至红细胞压积>30％或者HGB>100 g/L后应考虑减量。若使用推荐剂量治疗3～4周仍未达到上述疗效，建议考虑二线用药。急性重型AIHA可能需要使用100～200 mg/d甲泼尼龙10～14 d才能控制病情。

**图1:**
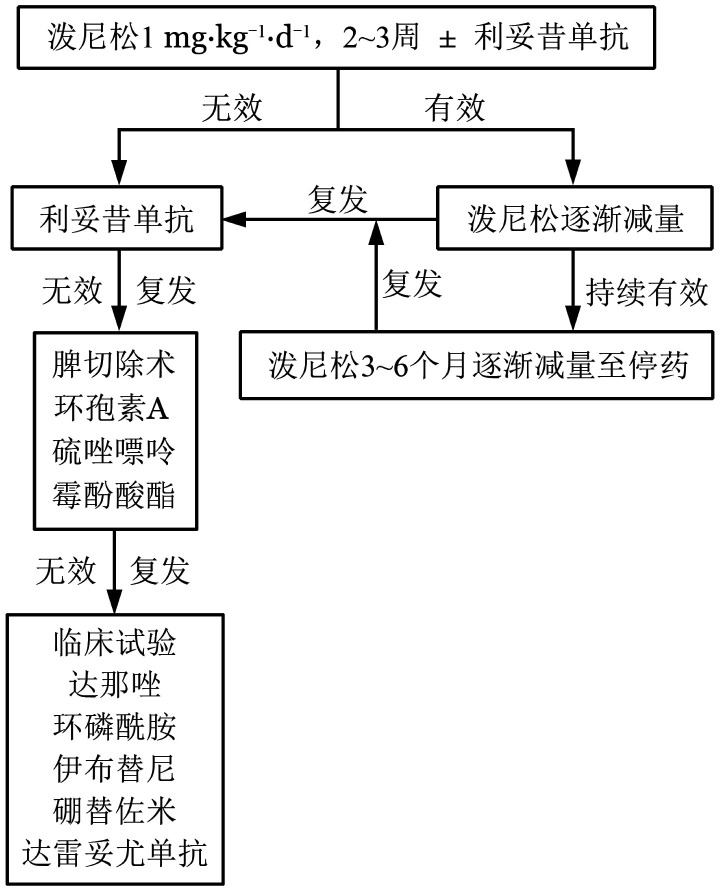
成人温抗体型自身免疫性溶血性贫血诊治流程图

有效者泼尼松剂量在4周内逐渐减至20～30 mg/d，以后每月递减（减少2.5～10.0 mg/d），在此过程中严密检测HGB水平和网织红细胞绝对值变化。泼尼松剂量减至5 mg/d并持续缓解2～3个月，考虑停用糖皮质激素。

激素耐药：泼尼松1 mg/kg及以上剂量，治疗3周无效。

激素依赖：需要泼尼松10 mg/d以上剂量才能维持疗效。

②糖皮质激素联合利妥昔单抗：目前有两个随机对照临床试验证实糖皮质激素联合利妥昔单抗一线治疗wAIHA疗效高于单用糖皮质激素，不良反应没有增加。第一个临床试验纳入64例wAIHA患者，随机分为单用泼尼松组和泼尼松联合利妥昔单抗组，利妥昔单抗375 mg·m^−2^·d^−1^，每周1次，连续4周。联合组12个月的有效率明显高于单药组（75％和36％），36个月无复发生存率也明显高于单药组（70％和45％）[10]。第二个临床试验采用固定剂量利妥昔单抗（1 000 mg，d 1、15）联合泼尼松一线治疗wAIHA，12个月和24个月的有效率都明显优于泼尼松单药（75％和31％、63％和19％）[11]。对于重度贫血或不适合大剂量糖皮质激素的AIHA患者，一线治疗可以选择糖皮质激素联合利妥昔单抗的方案。

**图2:**
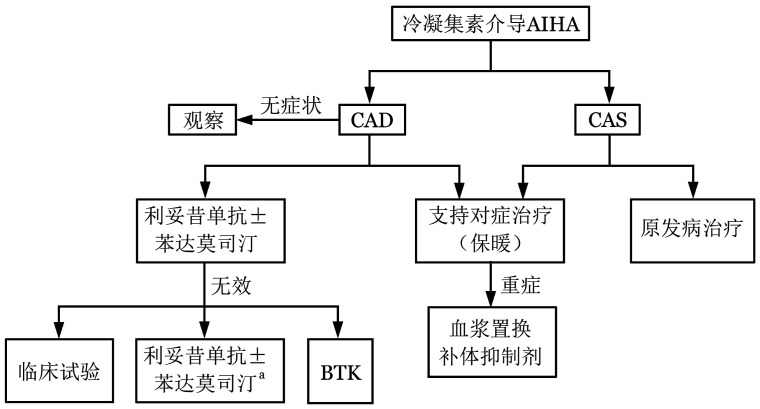
成人冷抗体型自身免疫性溶血性贫血（cAIHA）诊治流程图 注 CAD：冷凝集素病；CAS：冷凝集素综合征；BTK：布鲁顿激酶抑制剂。^a^：如果一线为利妥昔单抗单药治疗

（2）二线治疗：糖皮质激素治疗无效、复发、不耐受和依赖的患者都可以进行二线治疗。

二线治疗的首选方案是利妥昔单抗。如果一线治疗应用糖皮质激素联合利妥昔单抗无效或者短期内（<12个月）复发患者，直接进入三线治疗。

利妥昔单抗：利妥昔单抗二线治疗wAIHA的有效率79％左右[12]。利妥昔单抗的应用剂量有三种方案：标准剂量、固定大剂量和小剂量。标准剂量方案：利妥昔单抗375 mg·m^−2^·d^−1^，第1、8、15、22天，共4次[12]–[15]。固定大剂量方案：利妥昔单抗1 000 mg/d，第1、15天，共两次[11]。小剂量方案：利妥昔单抗100 mg/d，第1、8、15、22天，共4次[16]–[18]。目前标准剂量的临床试验数据最多，三个剂量方案有效率和不良反应无明显差异。利妥昔单抗的并发症包括感染、进行性多灶性白质脑病等，监测B淋巴细胞和免疫球蛋白水平可以指导控制。HBV感染患者应在抗病毒药有效控制并持续给药的情况下使用利妥昔单抗。

（3）三线治疗：三线治疗有脾切除和细胞毒性免疫抑制剂等。

①脾切除：对于难治性wAIHA，可考虑脾切除，有效率70％左右，完全缓解（CR）率为40％。尚无指标能预测脾切除的疗效。脾切除后感染发生率增加，尤其是致命重度感染的发生率明显增加，有条件的患者脾切除2周前可行疫苗接种（流感嗜血杆菌、脑膜炎奈瑟菌、肺炎球菌）。其他并发症有静脉血栓、肺栓塞、肺动脉高压等[19]–[20]。

②免疫抑制剂：最常用的有环孢素A、西罗莫司、硫唑嘌呤、霉酚酸酯等，一般有效率为40％～60％，多数情况下需要与糖皮质激素联用。

环孢素A治疗AIHA已经较广泛应用，多以3 mg·kg^−1^·d^−1^起给药，维持血药浓度（谷浓度）不低于150～200 µg/L。环孢素A不良反应有齿龈/毛发增生、高血压、胆红素增高、肾功能受损等。由于环孢素A需要达到有效血药浓度后才起效，建议初期与糖皮质激素联用[21]–[23]。

西罗莫司：对于难治/复发、实体器官移植或造血干细胞移植后或肾功能差的AIHA患者疗效较好，常用剂量1～2 mg/d，不良反应有高脂血症、血小板减少、口腔溃疡等。

硫唑嘌呤：常用剂量为2～2.5 mg·kg^−1^·d^−1^，不良反应包括骨髓抑制和肝脏毒性等，建议50 mg/d小剂量起始，耐受情况下，逐渐加到常用剂量。

霉酚酸酯：有效率报道不一，为25％～100％。建议500 mg每日两次起始。可加至1 000 mg每日两次。不良反应包括头痛、恶心、腹泻等。

（4）其他治疗药物

环磷酰胺：口服环磷酰胺常用剂量为50～100 mg/d，静脉环磷酰胺剂量为每次500～1 000 mg。有效率为50％～70％。起效时间为2～6周。常见不良反应包括骨髓抑制、感染、继发肿瘤、致畸性、不孕、泌尿系毒性等。多用于重症、伴有结缔组织病或淋巴细胞增殖性疾病（LPD）。

达那唑：常用剂量为200 mg每天3次。多需与糖皮质激素联用，有效率为50％～70％。常见不良反应包括肝脏毒性、男性化、前列腺肿瘤等。

硼替佐米：硼替佐米常用剂量为1.3 mg·m^−2^·d^−1^每周1次连续4次或第1、4、8、11天给药。有效率为31％～75％。主要不良反应包括神经毒性、骨髓抑制、腹泻、便秘等。

（5）继发性wAIHA

①慢性淋巴细胞白血病（CLL）相关wAIHA：根据CLL分期确定治疗方案。无CLL治疗指征者，治疗同原发性wAIHA；有CLL治疗指征者，选用CLL治疗方案。少数患者CLL治疗过程中会出现溶血加重，可联合糖皮质激素，必要时CLL药物减量或停药。待溶血控制后，再缓慢加用CLL治疗药物。常用药物包括利妥昔单抗、布鲁顿激酶抑制剂（伊布替尼）、维奈克拉、苯达莫司汀及联合方案。

②系统性红斑狼疮（SLE）相关wAIHA：治疗原则同原发性wAIHA。一线治疗是糖皮质激素；二线治疗包括利妥昔单抗、霉酚酸酯和硫唑嘌呤。

③普通变异型免疫缺陷病（CVID）相关wAIHA：患者感染率高，尤其是应用糖皮质激素、免疫抑制剂和单克隆抗体如利妥昔单抗等后，容易发生严重感染，甚至危及生命。治疗过程中，推荐给与静脉免疫球蛋白升高免疫球蛋白水平。一线治疗可以选用糖皮质激素，一旦有效后，迅速减量停药。避免长期大量应用糖皮质激素。二线治疗推荐利妥昔单抗[24]。

3. cAIHA的治疗

（1）一线治疗：

①利妥昔单抗单药：利妥昔单抗375 mg/m^2^，每周1次，连续4次。有效率50％左右，但CR率较低（3％）。有效者HGB可升高40 g/L。疗效持续时间少于1年[25]。复发后，再次应用多有效。不良反应多轻微。

②利妥昔单抗联合苯达莫司汀：利妥昔单抗375 mg/m^2^，第1天；苯达莫司汀70～90 mg·m^−2^·d^−1^，第1、2天。28 d为1个疗程。共4个疗程。有效率为71％，其中CR率为40％。CR者HGB升高44 g/L，部分缓解（PR）者HGB升高39 g/L。中位起效时间1.9个月。疗程持久，中位随访32个月，复发率仅9％。主要不良反应是粒细胞减少和发热等[26]。联合方案主要用于病情危重患者。

（2）二线治疗：

如果一线治疗方案是利妥昔单抗单药，无效和复发后，可以选择利妥昔单抗联合苯达莫司汀治疗。如果1年后复发，也可选择利妥昔单抗单药治疗。

如果一线治疗方案是利妥昔单抗联合苯达莫司汀，无效和短期复发（1年内），可以选择临床试验或者伊布替尼。如果2年后复发，也可选择再次应用利妥昔单抗联合苯达莫司汀治疗[27]。

伊布替尼：13例CAD/CAS患者应用伊布替尼（420 mg/d）治疗，有效率为100％，其中12例CR，1例PR。HGB中位升高56 g/L。全部患者3个月内脱离输血。多数患者1个月内起效，贫血和溶血减轻，雷诺现象缓解[28]–[29]。不良反应为轻度皮肤瘀斑、腹泻和皮疹等。

利妥昔单抗联合氟达拉滨：利妥昔单抗375 mg/m^2^，第1天；氟达拉滨25 mg·m^−2^·d^−1^，第1～5天。四周为1个疗程，共4个疗程。有效率为76％，其中CR率为21％，PR率为55％。有效者HGB中位升高31 g/L。中位起效时间4个月，中位疗效持续时间66个月。主要不良反应是骨髓抑制和感染等。联合治疗未发生单药氟达拉滨导致的wAIHA[30]。

硼替佐米：硼替佐米常用剂量为1.3 mg/m^2^，每周1次，连续4次。有效率为31％。主要不良反应包括神经毒性、骨髓抑制、腹泻、便秘等。

（3）三线治疗：补体抑制剂，补体C1抑制剂苏替利单抗（Sutimlimab）[31]、C3抑制剂Pegcetacoplan和C5抑制剂依库丽单抗（Eculizumab）能迅速控制部分CAD/CAS患者溶血发作，改善贫血。

（4）其他药物和治疗方法：静脉免疫球蛋白对部分AIHA患者有效。血浆置换对IgM型冷抗体效果较好（37 °C时80％ IgM型抗体呈游离状态），但对其他吸附在红细胞上温抗体效果不佳，且置换带入大量补体。

（5）继发性cAIHA治疗：多数cAIHA是继发性，需要积极寻找原发疾病，原发疾病的治疗根据相关疾病的指征[32]。保温对于cAIHA非常重要。

4. mAIHA的治疗

mAIHA的病情重，容易复发。一线治疗推荐糖皮质激素联合利妥昔单抗。糖皮质激素单药治疗有效率70％左右，但疗效不持久，容易复发，67％的患者需要二线治疗[33]。

5. PCH的治疗

PCH多可自限。如果贫血症状明显，可以输血。输血时应注意保温。补体抑制剂对急性溶血可能有效。

四、AIHA疗效标准

1. 痊愈：继发于感染者，在原发病治愈后，AIHA也治愈。无临床症状、无贫血、DAT阴性。CAS者CA效价正常。PCH者冷热溶血试验阴性。

2. CR：临床症状消失，HGB水平和网织红细胞百分比均正常，血清胆红素、结合珠蛋白和乳酸脱氢酶水平正常。CAD患者CR标准还包括：检测不到克隆性B细胞和克隆性IgM。

3. PR：HGB升高>20 g/L，或者HGB恢复正常但溶血生化指标未完全正常（包括网织红细胞、结合珠蛋白、胆红素和乳酸脱氢酶），脱离输血至少7 d。

4. 无效：未达到PR的标准。
